# Are We Speaking the Same Language? Terminology Consistency in EBD

**DOI:** 10.1177/19375867231225395

**Published:** 2024-01-24

**Authors:** Tahere Golgolnia, Maja Kevdzija, Gesine Marquardt

**Affiliations:** 1 Social and Health Care Buildings and Design, Faculty of Architecture, Technische Universitaet Dresden, Germany; 2 Institute of Architecture and Design, Faculty of Architecture and Planning, TU Wien, Austria

**Keywords:** evidence-based design, dementia-friendly design, architectural variables, health and care outcomes, terminology analysis, standardized terminology, frequency analysis, statistical tests

## Abstract

**Objective:**

The aim of this study is to analyze the consistency, variability, and potential standardization of terminology used to describe architectural variables (AVs) and health outcomes in evidence-based design (EBD) studies.

**Background:**

In EBD research, consistent terminology is crucial for studying the effects of AVs on health outcomes. However, there is a possibility that diverse terms have been used by researchers, which could lead to potential confusion and inconsistencies.

**Methods:**

Three recent large systematic reviews were used as a source of publications, and 105 were extracted. The analysis aimed to extract a list of the terms used to refer to the unique concepts of AVs and health outcomes, with a specific focus on people with dementia. Each term’s frequency was calculated, and statistical tests, including the χ^2^ and the post hoc test, were employed to compare their distributions.

**Results:**

The study identified representative terms for AVs and health outcomes, revealing the variability in terminology usage within EBD field for dementia-friendly design. The comparative analysis of the identified terms highlighted patterns of frequency and distribution, shedding light on potential areas for standardization.

**Conclusions:**

The findings emphasize the need for standardized terminologies in EBD to improve communication, collaboration, and knowledge synthesis. Standardization of terminology can facilitate research comparability, enhance the generalizability of findings by creating a common language across studies and practitioners, and support the development of EBD guidelines. The study contributes to the ongoing discourse on standardizing terminologies in the field and provides insights into strategies for achieving consensus among researchers, practitioners, and stakeholders in health environmental research.

## Introduction

Evidence-based design (EBD) is a field of research that has been attracting significant attention over the years, particularly in the healthcare field, due to its potential to improve health outcomes in the built environment (Mahmood, 2021). EBD studies have been conducted to investigate how the built environment can affect the physical and psychological health of its users (Evans, 2003). In the past few decades, numerous studies related to EBD and healthcare have been published, examining various aspects of how the built environment impacts its users. One important area of study has been the effect of healthcare facilities on people with dementia, which is a subset of EBD known as dementia-friendly design. This field focuses on improving the built environment for people with dementia (Brambilla et al., 2019; Marquardt et al., 2014; Waller & Masterson, 2015). It is important to note that among healthcare facilities, the research base of dementia-friendly design is more robust in long-term care facilities than in other types of healthcare facilities such as acute care (Hebert & Scales, 2019). This is due to the fact that in most countries, more than 80% of people in long-term care facilities (e.g., nursing homes) have dementia (Sanford et al., 2015).

The increase in dementia-friendly design studies in recent years as part of EBD has led to a fragmentation of knowledge, by introducing diverse methodologies, terminologies, and contextual variations, making it difficult to apply the results to practical design solutions (Gan et al., 2022; Marquardt et al., 2014). It is crucial to organize and consolidate the knowledge gained from these studies to create a more comprehensive understanding of how the built environment can be designed to support people with dementia. This effort could lead to a more coherent and effective approach to designing healthcare facilities for people with dementia, ultimately improving their health and quality of life.

***It is crucial to organize and consolidate the knowledge gained from these studies to create a more comprehensive understanding of how the built environment can be designed to support people with dementia.***

Dementia-friendly design is a multidisciplinary field that involves experts from a range of disciplines. Architects and designers play a crucial role in creating environments that support people with dementia, researchers contribute to the understanding of how design features can support health outcomes, medical specialists and caregivers provide valuable insights into the needs of people with dementia (Røsvik & Rokstad, 2020). Gerontologists provide insights into the aging process and how it relates to the needs of people with dementia (Day et al., 2000), and psychologists offer valuable input by examining the cognitive and emotional aspects of the built environment and how it affects people with dementia (Oatley & Johnson-Laird, 2014). Collaboration among these professionals is crucial in the development and implementation of dementia-friendly design strategies. Moreover, policy makers and healthcare providers can have a significant impact on the adoption and implementation of such strategies in healthcare facilities (Lin, 2017). As a result, by working together, these experts can create more effective and inclusive environments for people with dementia.

The multidisciplinary nature of dementia-friendly design highlights the criticality of having a common language and agreed-upon terminology. However, researchers in EBD, especially in dementia-friendly design, have utilized diverse terms to describe the architectural variables (AVs) and health and care outcomes (HCOs) when investigating the relationship between the built environment and health outcomes. Furthermore, the use of a wide range of terminology in health environments research has resulted in some ambiguity in the interpretation of terms (Battisto et al., 2023). This poses a significant challenge, as consistent and standardized terminology is crucial for effective communication and understanding among the various specialists involved in EBD (Anåker et al., 2017; Peavey & Vander Wyst, 2017).

***The multidisciplinary nature of dementia-friendly design highlights the criticality of having a common language and agreed-upon terminology.***

There is widespread agreement among researchers who specialize in standardized terminologies that a wide variety of inconsistent and diverse terminologies hinder the conduct of evidence syntheses, communication, collaboration, and knowledge of research findings in diverse settings. Standardized terminologies are particularly important in the healthcare industry and health environmental research, as they have the potential to improve patient care and safety (Battisto et al., 2023; Fennelly et al., 2021; World Health Organization, 2006). The use of standardized terminologies in clinical practice can enhance communication, quality of care, and interoperability and facilitate value-based healthcare and knowledge generation (Fennelly et al., 2021). For example, an international working group was established in 2012 to explore the development of a common terminology and a comprehensive framework for knowledge translation interventions that integrate evidence into health practices, systems, and policies (Colquhoun et al., 2014).

***There is widespread agreement among researchers who specialize in standardized terminologies that a wide variety of inconsistent and diverse terminologies hinder the conduct of evidence syntheses, communication, collaboration, and knowledge of research findings in diverse settings.***

Standardized terminology is also critical to the development and access of nursing knowledge. Without consistent terminology, the nursing knowledge base may be hindered, thereby delaying the integration of evidence-based healthcare into nursing practice (Andrews et al., 2016; Hardiker, 2011). Several pervious efforts have focused on the importance and effectiveness of standardized nursing terminologies for nursing practice and healthcare outcomes (Andrews et al., 2016; De Groot et al., 2020; Strudwick & Hardiker, 2016; Tastan et al., 2014; Zhang et al., 2021). Furthermore, a literature review has provided evidence for the usefulness of standardized nursing terminologies in various fields of application (Törnvall & Jansson, 2017). Additionally, incorporating the input of nurses into the design process can further enhance the development and implementation of standardized terminologies that are relevant and useful to nursing practice.

To tackle these challenges, enhancing the uniformity of terminology can be an effective solution (De Groot et al., 2020; Fennelly et al., 2021; Hardiker, 2011; Törnvall & Jansson, 2017; World Health Organization, 2006). Furthermore, there is growing recognition that standardized information can support statistical reporting, decision making, outcome and performance measurement, and cost analysis. Therefore, various dimensions of terminology must be accurately captured and standardized (World Health Organization, 2006). Recognizing the potential limitations of a purely positivist view on the relationship between the built environment and human outcomes, it is important to acknowledge the diverse perspectives and nuances that exist in the field. While this article aims to analyze the consistency and variability of terminology in EBD studies, it recognizes the need to consider a broader research paradigm encompassing qualitative and mixed-methods approaches to capture the complexity of the relationship.

In light of the aforementioned considerations, this article addresses the following question:

What are the most commonly used terms to describe the AVs of healthcare facilities and the HCOs of people with dementia in EBD studies?

To answer this question, this article examines the terms used in empirical research on the impact of AVs on HCOs within the context of dementia-friendly design through a systematic review. The goal is to identify commonly used terms, evaluate their consistency and variability across studies, and provide recommendations for standardizing terminology. Dementia-friendly design is a well-investigated topic in the field of EBD which provides a suitable context for investigating the commonly used terms to describe the AVs of healthcare facilities and the HCOs of people with dementia.

## Material and Methodology of Terminology Analysis

**Figure 1.**
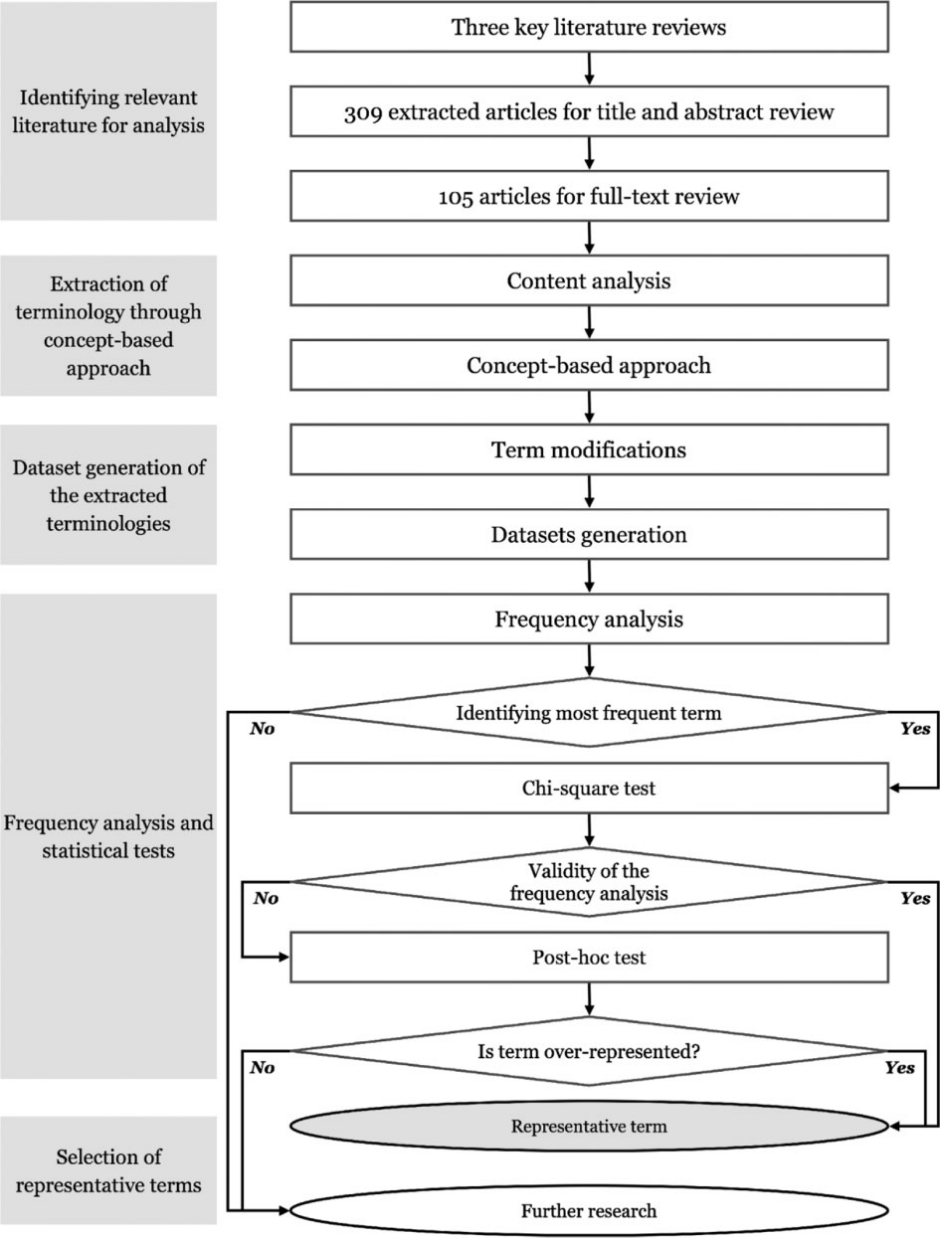
Schematic flowchart of the methodology sections.

The methodology for identifying representative terms consists of four main sections: identifying relevant empirical studies, extracting terms through a concept-based approach, generating datasets with term modifications, and conducting frequency analysis with statistical tests. The goal is to identify key concepts and provide a summary of the most commonly occurring and statistically significant terms. This is illustrated in Figure 1.

The search strategy for this review involved conducting a comprehensive search of relevant literature reviews related to EBD and dementia-friendly design. Three key recent literature reviews were selected and used for this study (listed below). Then, the previous reviews (105 studies) were retrieved for full-text review:

The Influence of the Physical Environment on Residents with Dementia in Long-Term Care Settings: A Review of the Empirical Literature (Chaudhury et al., 2018).
From Research to Application: Supportive and Therapeutic Environments for People Living with Dementia (Calkins, 2018).
Design and the Built Environment for People Living with Dementia in Residential Aged Care (Fleming et al., 2020).

The selection of three key recent literature reviews was based on specific criteria to ensure a comprehensive overview of dementia-friendly design in long-term care facilities. The chosen reviews, all conducted in 2018 or later, were considered the most recent to cover a spectrum of studies, including those featured in previous reviews and the latest publications. Additionally, the reviews were considered comprehensive as they addressed all aspects of dementia-friendly design, distinguishing them from those focusing solely on specific aspects. This selection process aimed to provide the audience with a robust and up-to-date synthesis of research in the field.

### Identifying Relevant Empirical Studies for Analysis

To identify relevant empirical studies for analysis, 309 references were retrieved from the three key systematic literature reviews. The title and abstract of each reference were screened based on inclusion and exclusion criteria. Studies were included if they investigated quantitatively the impact of AVs on HCOs for people with dementia, were published in English, and were conducted in long-term care settings or residential aged care facilities such as nursing homes. These types of healthcare facilities are well-investigated in the context of the impact of AVs on HCOs for people with dementia. This could mean that there is a larger body of research, consequently, more data available for analysis in these specific settings. After applying the criteria, 105 studies were selected for full-text review and critical evaluation of the terms applied to AVs and HCOs. Any duplicate studies were removed at this stage.

### Terminology Extraction Through a Concept-Based Approach

For terminology extraction, a content analysis was conducted to identify key concepts presented within the empirical studies. A concept-based approach was then applied, which involved identifying and categorizing terms based on their conceptual relevance and relationship to each other. This approach ensured that relevant terms were identified and analyzed in the context of the broader themes and concepts within the literature.

### Conducting Content Analysis of Selected Studies

To conduct the content analysis, each of the 105 selected studies was reviewed in detail to identify relevant terms related to the AVs and HCOs investigated. These terms were extracted and recorded in a spreadsheet for further analysis. The content analysis was conducted in two steps: first, each study was reviewed to identify terms used to describe the AVs and HCOs, and these terms were recorded alongside a unique study code. Second, the extracted terms were organized into categories based on their conceptual similarity.

### Application of a Concept-Based Approach

A concept is a general idea or category that encompasses specific instances or variations. For example, the concept of “light level” includes specific instances such as “amount of light,” “indoor light levels,” or “light.” This approach involved identifying the main concepts related to AVs and HCOs examined in the reviewed studies and compiling a list of different terms used by researchers to refer to each concept. Two separate tables were created for AVs and HCOs, listing the concepts along with their corresponding definitions (The list of concepts is presented in Tables 3 and 5, and the list of concepts with their definitions can be found in Tables A2 and A3 in the Supplementary Material). For each concept, a definition and a compilation of various terms used across different studies were established. The concepts were organized into different clusters and presented accordingly. As an illustration, Table 1 demonstrates the cluster of performance-focused concepts for AVs and their corresponding definitions.

**Table 1.** Performance-Focused Concepts for Architectural Variables and Their Corresponding Definitions.

Concept	Definition
Light level	The level of brightness or illumination within a space
Bright light exposure	The amount or duration of exposure to bright light in a space
Bright light source	The source or origin of bright light in a space
Daylight control	The ability to regulate the amount of natural light entering a space
Visual cue	The visual marker or signal used to guide or direct behavior or movement within a space
Physical cue	The physical marker or signal used to assist or direct behavior or movement within a space
Visual barrier	The visual obstruction that impedes visibility within a space
Physical barrier	The physical obstruction or boundary that physically separates different areas or zones within a space

### Dataset Generation of the Extracted Terms

To create datasets of the extracted terms related to AVs and HCOs, a review process was conducted to refine and clarify the terminology. This review revealed certain sources of variability in terminology. Several modifications were made to the extracted terms to obtain corresponding pure terms to standardize the scope of each concept and reduce variation. Three primary modifications were employed: replacing plural forms with singular forms, using the redundancy argument (i.e., eliminating terms that were found to be redundant or overlapping), and applying the variable-value distinction.

The use of singular and plural forms of terms was recognized as a source of variability. To reduce this variation, plural forms were converted to their corresponding singular forms (e.g., “outdoor environments” was changed to “outdoor environment”). Redundancy refers to the use of multiple terms or phrases to describe the same concept. In the reviewed studies, researchers may have used different words or phrases to describe a particular concept related to AVs and HCOs. To remove redundancy, for instance, the term “salient environmental cues” was simplified to “environmental cue,” while “assisted devices used” was changed to “assisted device.” Another example is the streamlining of “supportive wayfinding features with information necessary for the decision-making process” to “wayfinding feature.”

Another source of variability was the use of variables versus values to describe key concepts. Therefore, a variable-value distinction approach for each concept was applied to enable comparing and synthesizing findings across studies. In empirical research, “variable” refers to an attribute or characteristic that can vary across individuals or contexts. Variables refer to concepts that can change or vary, whereas values refer to specific observations or measurements of those variables (Babbie, 2016; Creswell, 2014; Maxwell, 2012). In this study, the concepts of AVs and HCOs can be considered variables. For example, the concept of “level of light” is a variable because it can be measured using a standardized metric and can vary depending on factors such as the time of day and the location of the light source. On the other hand, a “value” refers to a specific level of a variable, representing a particular measurement at a given point in time or space. Thereby, “high level of light” is a value because it represents a specific level of its variable. Values can also be classified into nominal values (those that represent categories or labels) and ordinal values (those that represent a rank order or hierarchy; Shreffler & Huecker, 2023).

### Frequency Analysis Along with Statistical Tests

In the previous sections, the terms for AVs and HCOs have been extracted. Then, through previously described modifications, a dataset of pure terms was generated. This dataset includes a list of terms for each concept and the number of times that researchers used a term in the reviewed studies. Table 2 shows the dataset of pure terms for the concept of “light level” as an example.

**Table 2.** Dataset of Pure Terms for the Concept of “Light Level.”

Pure Term	Count
Lighting	6
Light	3
Indoor light level	1
Amount of light	1
Intensity of light	1
Lighting in the dining areas	1
Average light level	1
Ambient light level	1

In this table, the “pure term” column lists the individual terms that researchers used to refer to the concept of “light level,” and the “count” column shows the number of times each term was used in the reviewed studies. The table provides a clear and concise way to organize the data and compare the usage of different terms for each concept.

The table is used as a basis for conducting a frequency analysis to identify the most commonly used term for each concept in the reviewed studies. However, relying solely on the observed frequency count is not enough to determine the representative term, as it may be due to chance. Statistical tests are necessary to confirm the results of the frequency analysis. The process involves calculating the observed frequency, relative frequency, and expected frequency of each term, followed by conducting a chi-square (χ^2^) test to determine if the observed frequencies differ significantly from the expected frequencies.

In this study specifically, the χ^2^ goodness-of-fit test is used to assess whether the observed frequency distribution of terms differed significantly from the expected distribution. Specifically, to determine whether any terms are significantly more or less frequent than others within the concept, a χ^2^ goodness-of-fit test with the following hypotheses is performed:

**Hypothesis 0:** There is no significant difference between the observed and expected frequencies of the terms for “concept.”
**Hypothesis A:** There is a significant difference between the observed and expected frequencies of the terms for “concept.”

Using the observed and expected frequencies, the χ^2^ test statistic is calculated for each concept as follows:


1 $$\chi^{2}\;=\;\sum \;{\left(\text{OF }-\text{ EF}\right)}^{2}/\text{ EF}$$

where OF is the observed frequency and EF is the expected frequency.

Then, the χ^2^ test is used to determine if there is a significant difference between the observed and expected frequencies of each term, and the null hypothesis is either accepted or rejected based on the calculated χ^2^ value and the critical value at a significance level of 0.05. When a significant difference is identified, post hoc tests such as residual analysis are conducted to investigate which specific terms are contributing the most to the significant difference.

The residual analysis, specifically Pearson’s residual, is used to identify which terms are significantly over- or underrepresented in the dataset, aiding in the interpretation of the results. This analysis helps to determine which specific terms have the largest discrepancies between observed and expected frequencies. In this study, the residual analysis was conducted to determine which terms had the most significant discrepancies between observed and expected frequencies in cases where the χ^2^ test showed a significant difference.

By using Pearson’s residual, which is a common method for residual analysis in contingency tables, as well as calculating the critical value using the following formulas, the post hoc test has been applied:


2 | Pearson’s residual | = | OF − EF | / sqrt (EF)

3 Critical value= z(α/2)×sqrt (OF)

In this formula, α represents the level of significance (significance level of 0.05), where z(α/2) is the *z*-score for the desired level of significance (α/2) and sqrt (OF) is the square root of 2 for the observed frequency.

The z-score is a statistical measure that indicates how far an observation is from the mean of a distribution in terms of standard deviations. It is calculated by subtracting the mean from the dataset and dividing the result by the standard deviation. The formula for the z-score is:


4 z = (OF−mean) / SD

where OF is the observed frequency, mean is the mean of the distribution, and *SD* is the standard deviation of the distribution. By utilizing the above formulas, where the absolute value of Pearson’s residual for a term is greater than the critical value, that term is considered to be significantly different from what would be expected by chance and may be contributing to the overall significant χ^2^ result. In the following, the results of frequency analysis along with statistical tests that led to the selection of representative terms are presented separately for the concepts of AVs and HCOs. However, for some other AVs and HCOs, it was not feasible to introduce the representative term based on the available datasets. Therefore, further research has been suggested for those concepts.

## Results

### Representative Terms for the Concepts of AVs

Table 3 presents a list of representative terms for the concepts of AVs. This table was developed based on the selection criteria of frequency analysis, the χ^2^ test, and a comparison with the critical values for each concept.

**Table 3.** Selection of Representative Terms for Architectural Variables.

ID	Concept	Term With Highest Frequency	Highest Relative Frequency (%)	OF	EF	Degree of Freedom	Chi-Square	Critical Value	Chi-Square Is Less Than Critical Value
1	Area	Size	67	6	2.25	3	8.33	7.815	No
2	Spatial density/capacity	Number of residents	29	8	2.15	12	24.93	21.026	No
3	Count of spaces	—							
4	Floor covering color	Color contrast between table and floor	100	2	2	1	0	3.841	Yes
5	Table setting color contrast	—							
6	Floor covering material	—							
7	Outdoor space	Outdoor area	18	4	1.47	14	8	23.685	Yes
8	Kitchen	Meal preparation	33	2	1.2	4	0.667	9.488	Yes
9	Dining space	Dining room	43	9	3.5	5	15.286	11.07	No
10	Common space	Living area	19	4	1.75	11	7	19.675	Yes
11	Bedroom	Bedroom	100	4	4	3	0	7.815	Yes
12	Family space	—							
13	Medical space	—							
14	Personal hygiene space/toilet	Toilet	100	3	3	2	0	5.991	Yes
15	Personal hygiene space/bathroom	—							
16	Staff space	—							
17	Circulation system/spatial configuration	—							
18	Visual connection	Visual access	36	4	1.57	6	4.909	12.592	Yes
19	Physical connection	—							
20	Indoor temperature	—							
21	Noise level	Noise level	44	4	2.25	3	3	7.815	Yes
22	Noise source	—							
23	Furniture type	Furniture	38	5	1.625	7	8.538	14.067	Yes
24	Furniture character/style	—							
25	Light level	Lighting	40	6	1.875	7	12.2	14.067	Yes
26	Bright light exposure	—							
27	Bright light source	Lighting system	80	4	2.5	1	1.8	3.841	Yes
28	Daylight control	—							
29	Visual cue	Environmental cue	15	2	1.083	11	0.846	19.675	Yes
30	Physical cue	Physical support	33	2	1.2	4	0.667	9.488	Yes
31	Visual barrier	—							
32	Physical barrier	Safety	33	6	1.8	9	13.111	16.919	Yes
33	Possibility of personalizing the environment	—							
34	Food service	Food service	50	2	1.33	2	0.5	5.991	Yes
35	Gradation of space	—							
36	Visual elements	Visual stimulation	33	3	1.29	6	2.667	12.592	Yes
37	Auditory elements	—							
38	Tactile elements	—							
39	Olfactory elements	—							
40	Overall ambiance atmosphere	—							

Table 3 presents 40 concepts related to AVs, many of which have representative terms with a high frequency and a positive result in the χ^2^ test confirming the frequency analysis. However, despite having representative terms, three concepts showed a significant difference between observed and expected frequency according to the χ^2^ test. These three concepts were further analyzed through a post hoc analysis, and the results are presented in Table 4.

**Table 4.** Frequency Analysis and Post Hoc Test Results for Selected Concepts.

ID	Concept	Term With Highest Frequency	Highest Relative Frequency (%)	Critical Value Post Hoc Test	Pearson’s Residual	Pearson’s Residual Is More Than Critical Value
1	Area	Size	67	0.092	2.500	Yes
2	Spatial density/capacity	Number of residents	29	0.195	3.983	Yes
9	Dining space	Dining room	43	0.126	2.940	Yes

Table 4 presents the post hoc analysis results for three concepts: “area,” “spatial density or capacity,” and “dining space.” The results emphasize the importance of conducting post hoc tests to ensure the validity of the representative term. For example, the term with the highest frequency for the “area” concept was “size,” with a significant Pearson’s residual of 2.500. Similarly, the terms with the highest frequency for the “spatial density or capacity” and “dining space” concepts were “number of residents” and “dining room,” with significant Pearson’s residuals of 3.983 and 2.940, respectively.

### Representative Terms for the Concepts of HCOs

The list of representative terms for the HCOs’ concepts is presented in Table 5. This table was generated using selection criteria such as frequency analysis, the χ^2^ test, and a comparison with critical values for each concept.

**Table 5.** Selection of Representative Terms for Health and Care Outcomes.

ID	Concept	Term With Highest Frequency	Highest Relative Frequency (%)	Observed Frequency	Expected Frequency	Degrees of Freedom	Chi-Square	Critical Values	Chi-Square Is Less Than Critical value
1	Heart rate	–							
2	Blood pressure	–							
3	Body Temperature	–							
4	Physiological therapeutic care	–							
5	Mortality	–							
6	Physical therapeutic care	Physical restraint	13	4	1.19	25	8.419	37.652	Yes
7	Pain	Pain	75	3	2	1	1	3.841	Yes
8	Weight	Weight	63	5	2.67	2	3.25	5.991	Yes
9	Food intake	Food intake	12	3	1.14	21	4.04	32.671	Yes
10	Fluid intake	Fluid intake	50	2	1.33	2	0.5	5.991	Yes
11	Cognitive ability	Cognitive status	16	3	1.27	14	3.895	23.685	Yes
12	Cognitive impairment	Cognitive impairment	36	4	1.57	6	4.909	12.592	Yes
13	Circadian rhythm	Circadian rhythms	50	3	2	2	1	5.991	Yes
14	Sleep disturbance	Sleep	28	5	1.38	12	10.889	21.026	Yes
15	Psychotropic drug use	–							
16	Stimulation	Stimulation	31	4	1.63	7	6.077	14.067	Yes
17	Neuropsychiatric symptom	Anxiety	17	4	1.2	19	7.667	30.144	Yes
18	Aggressive behavior	Aggressive behavior	25	2	1.14	6	0.75	12.592	Yes
19	Disruptive behavior	Behavior difficulties	14	4	1.32	21	9.69	32.671	Yes
20	Agitated behavior	Agitation	20	6	1.67	17	20.4	27.587	Yes
21	Confused reaction behavior	–							
22	Social contact	Social interaction	23	5	1.47	14	10.727	23.685	Yes
23	Informal social interaction	Informal social interaction	18	2	1.1	9	0.818	16.919	Yes
24	Communication skill	–							
25	Engagement in activity	Proportion of time active	8	3	1.15	30	5.462	43.773	Yes
26	Activity of daily life	–							
27	Walking performance	Falls	21	3	1.17	11	3.143	19.675	Yes
28	Wayfinding	Wayfinding	14	3	1.57	13	3.455	22.362	Yes
29	Frequency of toilet use	–							
30	Motor and process test	Motor function	33	2	1.2	4	0.667	9.488	Yes
31	Privacy	Provision of privacy	33	2	1.2	4	0.667	9.488	Yes
32	Safety	Safety	27	3	1.57	6	2.364	12.592	Yes
33	Quality of life	Quality of life	46	21	2.71	11	134.348	19.675	No
34	Liberty and autonomy	–							
35	Personal strength	Sense of home	11	2	1.06	17	0.895	27.587	Yes

Table 5 displays 35 concepts associated with HCOs, where numerous concepts have representative terms with a high frequency and a positive χ^2^ test result that supports the frequency analysis. This table also shows that only one of the concepts, “quality of life,” has a significant χ^2^ test value, indicating that the frequency of the term “quality of life” is significantly higher than expected by chance. The post hoc test also confirmed that this term significantly contributed to the significant difference and is overrepresented in the data. Thus, it can be concluded that “quality of life” is the most frequent term for this concept.

## Discussion

The results of the study indicate that there are a variety of terms used in EBD and dementia-friendly design studies to refer to the concepts of AVs and HCOs. This research determined the most commonly used term for each unique concept of AVs and HCOs by selecting terms with the highest frequency. The frequency analysis and χ^2^ tests provided valuable insights into the terms used to describe AVs and HCOs in the revniewed studies to identify standardized terminologies. The representative terms identified in this study could serve as a valuable resource for future researchers. These standardized terminologies not only ensure consistency in future studies but also facilitate more effective communication and collaboration among researchers.

### Contribution to Efforts to Standardize Terminologies

In the field of EBD, there have been previous efforts to standardize terminology. One study critically examined and compared the conceptual meanings and underlying assumptions of EBD and research-informed design to facilitate practical use and theoretical development (Peavey & Vander Wyst, 2017). Ulrich and colleagues proposed a conceptual framework based on literature reviews (Ulrich et al., 2010). Additionally, the Center for Health Design’s Research Coalition initiated a project to create a standardized glossary of environmental terms and healthcare outcome measures. Phase I of this glossary development identified priority areas and common variables linking design elements to healthcare outcomes (Battisto et al., 2023; Quan et al., 2011). Another study aimed to increase awareness of the concept of “design quality” in healthcare architecture and resulted in a taxonomy of themes and terms used in the literature. This taxonomy provides a comprehensive understanding of how researchers, architects, and designers use the concept of design quality in healthcare architecture, with a focus on environmental sustainability, social and cultural interactions, and resilient construction. The study emphasizes the need for clear and explicit definitions of design quality to meet the complex needs of healthcare stakeholders (Anåker et al., 2017).

Just as Anåker et al. (2017) emphasized the need for standardized terminology in referring to design quality, the use of consistent terminology is also crucial in the emerging field of EBD in healthcare facilities, especially in relation to dementia-friendly design. By establishing standardized terminology, this effort aims to promote a more comprehensive and systematic approach to enhance the health and quality of life of people with dementia. One significant contribution of this standardization is facilitating easier interdisciplinary communication and the implementation of research findings into practice. Additionally, it provides a foundation for various practical applications, including the development of an assessment tool. An assessment tool can serve as a valuable resource for designing and evaluating the built environment, specifically in relation to the AVs that influence HCOs and their interrelationships (Calkins et al., 2022; Elf et al., 2017; Golgolnia et al., 2023; Mangili & Capolongo, 2022). The development of standardized terminology, as introduced in this study, lays the groundwork for further analysis, practical applications, and interdisciplinary collaboration in the field of EBD for dementia-friendly environments.

### Advantages of Using Variable-Value Distinction Approach

Previous research has employed the variable-value distinction approach to standardize terminology and enhance the consistency of data analysis. By employing pure variables without values, this approach minimizes errors in statistical analysis and enables researchers to concentrate on the core concepts being measured rather than specific measurement values. The findings of Gelman and Hill (2007) and Schwarz and Oyserman (2001) indicated that consistent terminology usage resulted in more reliable and accurate outcomes, particularly in the context of cross-cultural research.

The use of pure terms for variables without values allowed for greater flexibility in analyzing the data and provided a common language for researchers to describe and compare the various concepts related to AVs and HCOs. This approach also enables researchers to easily add new terms to the list of pure terms as new technologies and design innovations emerge in the field. The use of operational definitions, which clarify the meaning of key concepts and distinguish between variables and values, is essential for ensuring the reliability and validity of research findings (Babbie, 2016; Creswell, 2014). The variable-value distinction approach proved valuable in standardizing terminology and reducing variation in the language used to describe AVs and HCOs in the reviewed studies.

### Scenarios for Establishing Comprehensive and Standardized Terminology

**Figure 2.**
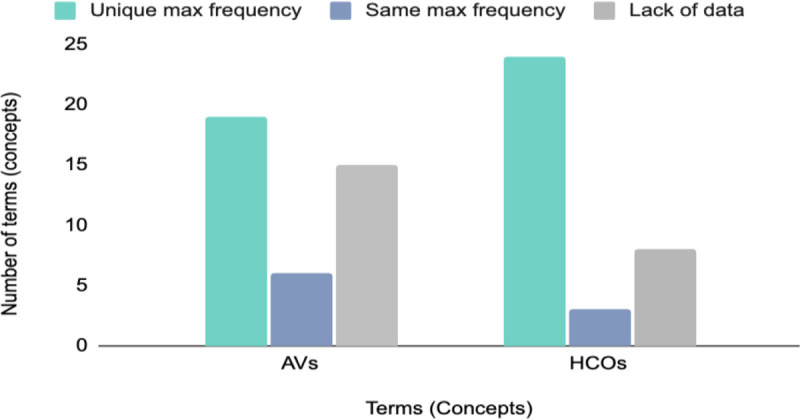
Distribution of frequency analysis results for architectural variables and health and care outcomes concepts.

Based on the result of frequency analysis in this article, Figure 2 in the following shows the number of concepts with unique highest frequencies, the same highest frequencies, and the concepts with the lack of data.

**Figure 3.**
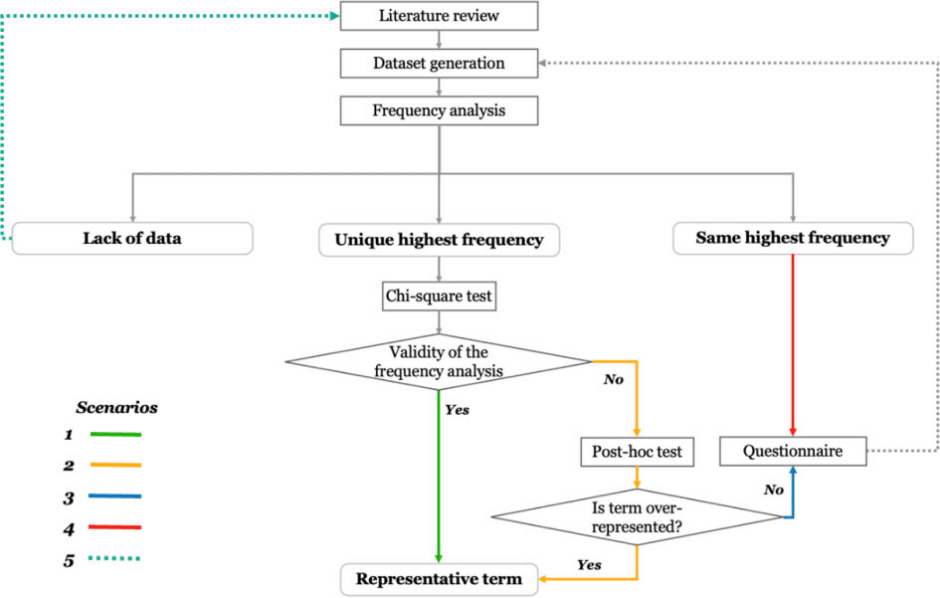
Proposed process for achieving comprehensive standardized terminology for architectural variables and health and care outcomes.

Although the majority of concepts in both AVs and HCOs groups have representative terms, Figure 2 shows a significant lack of data for some concepts, which is a challenge that needs to be addressed. To address this challenge and develop a comprehensive and standardized terminology for AVs and HCOs, the findings of this study can be used to inform future research. Figure 3 illustrates the entire process of developing a standardized terminology, which includes the part of the process done in this article and the part suggested for future research.

As Figure 3 illustrates, it is required to identify the representative terms for each concept based on their frequency of occurrence in the literature. However, some concepts may have more than one term with the same highest frequency. In such cases, it is proposed to collect additional data from experts’ opinions through a questionnaire and perform a second frequency analysis to determine the most representative term among the terms with the same frequency. Moreover, some concepts may not have enough data to conduct a frequency analysis in the first place. For these concepts, it is suggested to conduct the new literature review with a specific focus area (focusing on the specific concept or a specific cluster of concepts) and generate a new dataset that allows for frequency analysis and finding representative terms.

Figure 3 also outlines how to identify a representative term for each concept based on the frequency analysis conducted in this article. For this purpose, five scenarios can be considered for drawing conclusions based on the result of frequency analysis to identify the most representative term for a given concept.

The first scenario involves identifying a unique highest frequency. If the χ^2^ test confirms the validity of the frequency analysis, the representative term can be introduced without a post hoc test. A post hoc test is necessary in the second scenario if the χ^2^ test reveals a significant difference between the observed and expected frequencies. The third scenario suggests conducting further research using a questionnaire if the χ^2^ and post hoc tests do not confirm the frequency analysis result. The fourth scenario involves multiple terms sharing the same highest frequency, and experts’ opinions are recommended to identify a representative term. Lastly, in the fifth scenario, a lack of data prevents the identification of a representative term, and conducting a further literature review is suggested. In this case, conducting a review in medical literature could be a way to come across with the representative terminologies for HCOs. This process allows for a more robust and statistically rigorous analysis of the frequency distribution of terms for each concept, providing insight into the terms commonly used in the literature on the impact of AVs on HCOs for people with dementia.

The identification of representative terms for 40 AV concepts and 35 HCO concepts was carried out, suggesting the five-scenario process. The results of this process are summarized in Table 6 and 7 for AV findings and Table 8 and 9 for HCO findings.

**Table 6.** Overview of Frequency Analysis Results Across Architectural Variable Concepts With Unique Highest Frequency.

ID	Concept	Corresponding Representative Term
1	Area	Size
2	Spatial density/capacity	Number of residents
3	Floor covering color	Color contrast between table and floor
4	Outdoor space	Outdoor area
5	Kitchen	Meal preparation
6	Dining space	Dining room
7	Common space	Living area
8	Bedroom	Bedroom
9	Personal hygiene space/toilet	Toilet
10	Visual connection	Visual access
11	Noise level	Noise level
12	Furniture type	Furniture
13	light level	Lighting
14	Bright light source	Lighting system
15	Visual cue	Environmental cue
16	Physical cue	Physical support
17	Physical barrier	Safety
18	Food service	Food service
19	Visual elements	Visual stimulation

**Table 7.** Overview of Frequency Analysis Results Across Architectural Variable Concepts With Same Highest Frequency and Lack of Data.

ID	Concept	Frequency Analysis	Recommendation
1	Circulation system/spatial configuration	Same highest frequency	Questionnaire is recommended
2	Physical connection		
3	Indoor temperature		
4	Bright light exposure		
5	Possibility of personalizing the environment		
6	Overall ambiance atmosphere		
7	Count of spaces	Lack of data	Further research is recommended
8	Table setting color contrast		
9	Floor covering material		
10	Family space		
11	Medical space		
12	Personal hygiene space/bathroom		
13	Staff space		
14	Noise source		
15	Furniture character/style		
16	Daylight control		
17	Visual barrier		
18	Gradation of space		
19	Auditory elements		
20	Tactile elements		
21	Olfactory elements		

**Table 8.** Overview of Frequency Analysis Results Across Health and Care Outcome Concepts With Unique Highest Frequency.

ID	Concept	Corresponding Representative Term
1	Physical therapeutic care	Physical restraint
2	Pain	Pain
3	Weight	Weight
4	Food intake	Food intake
5	Fluid intake	Fluid intake
6	Cognitive abilities	Cognitive status
7	Cognitive impairment	Cognitive impairment
8	Circadian rhythms	Circadian rhythms
9	Sleep disturbance	Sleep
10	Stimulation	Stimulation
11	Neuropsychiatric symptoms	Anxiety
12	Aggressive behavior	Aggressive behavior
13	Disruptive behavior	Behavior difficulties
14	Agitated behavior	Agitation
15	Social contact	Social interaction
16	Informal social interactions	Informal social interaction
17	Engagement in activities	Proportion of time active
18	Walking performance	Falls
19	Wayfinding	Wayfinding
20	Motor and process test	Motor function
21	Privacy	Provision of privacy
22	Safety	Safety
23	Quality of life	Quality of life
24	Personal strengths	Sense of home

**Table 9.** Overview of Frequency Analysis Results Across Health and Care Outcome Concepts With Same Highest Frequency and Lack of Data.

ID	Concept	Frequency Analysis	Recommendation
1	Psychotropic drug use	Same highest frequency	Questionnaire is recommended
2	Activity of daily life		
3	Liberty and autonomy		
4	Heart rate	Lack of data	Further research is recommended
5	Blood pressure		
6	Body temperature		
7	Physiological therapeutic care		
8	Mortality		
9	Confused reaction behavior		
10	Communication skills		
11	Frequency of toilet use		

Among the AV and HCO concepts, in addition to the concepts that resulted in the unique highest frequency in which their representative terms are introduced, there were instances where multiple terms had the same highest frequency. In such cases, seeking expert input to determine a representative term is recommended. Additionally, there was insufficient data for certain AV and HCO concepts to conduct a comprehensive frequency analysis. In these instances, some concepts had terms with a frequency of 1 before and after term modification, while others had only one term identified after reviewing the studies. This lack of diversity in terminology poses challenges for conducting reliable frequency analysis and drawing meaningful conclusions. Therefore, further research with a specific and focused approach is necessary. This research should aim to gather additional terms and data, enabling the identification of representative terms and the establishment of standardized terminology.

Overall, the selection of representative terms based on frequency analysis is an essential step in this research that ensures the validity and reliability of the findings. By selecting representative terms, a clear understanding of the language used as AVs and HCOs in the reviewed studies is obtained, and the criteria used for selection provide transparency and reproducibility in future studies.

### Strengths and Limitations

This article has identified and addressed several important concepts and their associated terms related to AVs and HCOs. One of the key strengths of this study lies in its utilization of the variable-value distinction approach, which has proven valuable in standardizing terminology and reducing variation in the language used to describe AVs and HCOs across the reviewed studies. This approach has introduced representative terms, laying the groundwork for further analysis and practical application. Furthermore, this study contributes to the development of standardized terminologies by comprehensively analyzing the concepts, their frequency, and the statistical significance of their associated terms. This serves as one of the first steps toward promoting consistency and clarity in the field.

However, it is important to acknowledge the limitations of this study and address potential criticisms. The research paradigm adopted in this article leans toward a positivist perspective, primarily focusing on quantitative analysis of the relationship between the built environment and human outcomes. It is crucial to recognize that the relationship between the built environment and human outcomes can be nuanced and multifaceted, requiring a broader range of research approaches to capture its full complexity. While this study contributes to the advancement and standardization of terminology in the domain of EBD, it is essential to recognize the need for complementary qualitative research and a more comprehensive understanding of the subjective experiences and qualitative aspects related to AVs and HCOs.

Furthermore, this study has inherent limitations that should be considered. The subjective judgments involved in defining and labeling concepts may introduce bias or inconsistency in the analysis. The focus of this study is limited to dementia-friendly design, which may restrict the generalizability of the results to other contexts within the broader field of EBD. However, the framework and methodology presented in this article provide a foundation that can be expanded upon in future research to encompass a more diverse range of studies, allowing for a more comprehensive and inclusive understanding of terminology standardization in the field of EBD.

## Conclusion

This article provided a comprehensive analysis of the terms used in the EBD studies to refer to the concepts of AVs and HCOs in the dementia-friendly design field and to generate a list of representative terms for them through content and frequency analysis along with the statistical tests. The results of this study indicate that standardizing terminology related to AVs and HCOs is essential for effective communication and the comparison of findings across different studies. The majority of representative terms were easily identifiable, indicating general agreement among researchers in the field. However, it is important to acknowledge the need for further investigation in cases where the observed frequencies did not result in the introduction of representative terms. In such cases, questionnaires and specific area-focused systematic reviews can provide a more comprehensive understanding of the appropriate representative terms. The findings of this study contribute to the development of EBD research by providing a standardized terminology for the concepts of AVs and HCOs and a foundation for future research exploring the potential benefits of incorporating AVs into healthcare design to improve HCOs for people with dementia.

## Implications for Practice

- Standardizing terminology in EBD research can improve communication and collaboration among researchers, practitioners, and stakeholders.
- Using standardized terminology can facilitate the comparison of findings across different studies, enhancing the generalizability of results.
- The development of EBD guidelines can be supported by the standardization of terminology.
- Further investigation may be needed in cases where representative terms are not easily identifiable.
- Incorporating architectural variables into healthcare design can potentially improve health outcomes for people with dementia.

## Supplemental Material

Supplemental Material, sj-pdf-1-her-10.1177_19375867231225395 - Are We Speaking the Same Language? Terminology Consistency in EBD Supplemental Material, sj-pdf-1-her-10.1177_19375867231225395 for Are We Speaking the Same Language? Terminology Consistency in EBD by Tahere Golgolnia, Maja Kevdzija and Gesine Marquardt in HERD: Health Environments Research & Design Journal

## References

[bibr1-19375867231225395] Anåker A. Heylighen A. Nordin S. Elf M. (2017). Design quality in the context of healthcare environments: A scoping review. Health Environments Research & Design Journal, 10(4), 136–150. 10.1177/1937586716679404 28643560 PMC5484461

[bibr2-19375867231225395] Andrews J. C. Bogliatto F. Lawson H. W. Bornstein J. (2016). Speaking the same language: Using standardized terminology. Journal of Lower Genital Tract Disease, 20(1), 8–10. 10.1097/LGT.0000000000000157 26579837

[bibr3-19375867231225395] Babbie E. (2016). The practice of social research. Cengage Learning.

[bibr4-19375867231225395] Battisto D. Li X. Dong J. Hall L. Blouin J. (2023). Research methods used in evidence-based design: An analysis of five years of research articles from the HERD journal. Health Environments Research & Design Journal, 16(1), 56–82. 10.1177/19375867221125940 36424761

[bibr5-19375867231225395] Brambilla A. Rebecchi A. Capolongo S. (2019). Evidence based hospital design. A literature review of the recent publications about the EBD impact of built environment on hospital occupants’ and organizational outcomes. Annali Di Igiene: Medicina Preventiva E Di Comunita, 31(2), 165–180. 10.7416/ai.2019.2269 30714614

[bibr6-19375867231225395] Calkins M. P. (2018). From research to application: Supportive and therapeutic environments for people living with dementia. The Gerontologist, 58(suppl_1), S114–S128. 10.1093/geront/gnx146 29361065

[bibr7-19375867231225395] Calkins M. P. Kaup M. L. Abushousheh A. M. (2022). Evaluation of environmental assessment tools for settings for individuals living with dementia. Alzheimer’s & Dementia: Translational Research & Clinical Interventions, 8(1), e12353. 10.1002/trc2.12353 36204348 PMC9523676

[bibr8-19375867231225395] Chaudhury H. Cooke H. A. Cowie H. Razaghi L. (2018). The influence of the physical environment on residents with dementia in long-term care settings: A review of the empirical literature. The Gerontologist, 58(5), e325–e337. 10.1093/geront/gnw259 28329827

[bibr9-19375867231225395] Colquhoun H. Leeman J. Michie S. Lokker C. Bragge P. Hempel S. McKibbon K. A. Peters G.-J. Y. Stevens K. R. Wilson M. G. Grimshaw J. (2014). Towards a common terminology: A simplified framework of interventions to promote and integrate evidence into health practices, systems, and policies. Implementation Science, 9(1), 781. 10.1186/1748-5908-9-51 PMC4021969 24885553

[bibr11-19375867231225395] Creswell J. W. (2014). Research design: Qualitative, quantitative, and mixed methods approaches (4th ed). Sage. 10.7748/nr.12.1.82.s2 28718745

[bibr12-19375867231225395] Day K. Carreon D. Stump C. (2000). The therapeutic design of environments for people with dementia: A review of the empirical research. The Gerontologist, 40(4), 397–416. 10.1093/geront/40.4.397 10961029

[bibr14-19375867231225395] De Groot K. De Veer A. J. E. Paans W. Francke A. L. (2020). Use of electronic health records and standardized terminologies: A nationwide survey of nursing staff experiences. International Journal of Nursing Studies, 104, 103523. 10.1016/j.ijnurstu.2020.103523 32086028

[bibr15-19375867231225395] Elf M. Nordin S. Wijk H. Mckee K. J. (2017). A systematic review of the psychometric properties of instruments for assessing the quality of the physical environment in healthcare. Journal of Advanced Nursing, 73(12), 2796–2816. 10.1111/jan.13281 28207946

[bibr16-19375867231225395] Evans G. W. (2003). The built environment and mental health. Journal of Urban Health, 80(4), 536–555. 10.1093/jurban/jtg063 14709704 PMC3456225

[bibr17-19375867231225395] Fennelly O. Grogan L. Reed A. Hardiker N. R. (2021). Use of standardized terminologies in clinical practice: A scoping review. International Journal of Medical Informatics, 149, 104431. 10.1016/j.ijmedinf.2021.104431 33713915

[bibr18-19375867231225395] Fleming R. Zeisel J. Bennett K. (2020). World Alzheimer Report 2020: Design, dignity, dementia: Dementia-related design and the built environment. Retrieved September 21, 2020, from https://www.alzint.org/resource/world-alzheimer-report-2020/

[bibr19-19375867231225395] Gan D. R. Y. Chaudhury H. Mann J. Wister A. V. (2022). Dementia-friendly neighborhood and the built environment: A scoping review. The Gerontologist, 62(6), e340–e356. 10.1093/geront/gnab019 33564829

[bibr20-19375867231225395] Gelman A. Hill J. (2007). Data analysis using regression and multilevel/hierarchical models. Cambridge University Press.

[bibr21-19375867231225395] Golgolnia T. Kevdzija M. Marquardt G. (2023). Proposing a systematic assessment tool for evaluating the architectural variables of dementia-friendly design in nursing homes. In Goodman-Deane J. Dong H. Heylighen A. Lazar J. Clarkson J. (Eds.), Design for sustainable inclusion (pp. 59–69). Springer International. 10.1007/978-3-031-28528-8_7

[bibr42-19375867231225395] Hardiker N. R. (2011). Developing standardised terminologies to support nursing practice. In: McGonigle D Mastrian K (eds.), Nursing informatics and the foundation of knowledge, Second edition. Boston, USA: Jones and Bartlett Publishers, pp. 111–120.

[bibr22-19375867231225395] Hebert C. A. Scales K. (2019). Dementia friendly initiatives: A state of the science review. Dementia 18(5), 1858–1895. 10.1177/1471301217731433 28933191

[bibr23-19375867231225395] Lin S.-Y. (2017). ‘Dementia-friendly communities’ and being dementia friendly in healthcare settings. Current Opinion in Psychiatry, 30(2), 145–150. 10.1097/YCO.0000000000000304 27997454 PMC5287032

[bibr24-19375867231225395] Mahmood F. J. (2021). The role of evidence-based design in informing health-care architects. Journal of Facilities Management, 19(2), 249–262. 10.1108/JFM-09-2020-0062

[bibr25-19375867231225395] Mangili S. Capolongo S. (2022). Healthcare facilities and dementia development of a framework to assess design quality. Studies in Health Technology and Informatics, 297, 323–330. 10.3233/SHTI220856 36073410

[bibr26-19375867231225395] Marquardt G. Bueter K. Motzek T. (2014). Impact of the design of the built environment on people with dementia: An evidence-based review. Health Environments Research & Design Journal, 8(1), 127–157. 10.1177/193758671400800111 25816188

[bibr27-19375867231225395] Maxwell J. (2012). Qualitative research design: An interactive approach. Sage.

[bibr28-19375867231225395] Oatley K. Johnson-Laird P. N. (2014). Cognitive approaches to emotions. Trends in Cognitive Sciences, 18(3), 134–140. 10.1016/j.tics.2013.12.004 24389368

[bibr29-19375867231225395] Peavey E. Vander Wyst K. B. (2017). Evidence-based design and research-informed design: What’s the difference? Conceptual definitions and comparative analysis. Health Environments Research & Design Journal, 10(5), 143–156. 10.1177/1937586717697683 28349729

[bibr30-19375867231225395] Quan X. Malone E. Joseph A. Pati D. (2011). Healthcare environmental terms and outcome measures: An evidence-based design glossary. https://scholars.ttu.edu/en/publications/healthcare-environmental-terms-and-outcome-measures-an-evidence-b-5

[bibr31-19375867231225395] Røsvik J. Rokstad A. M. M. (2020). What are the needs of people with dementia in acute hospital settings, and what interventions are made to meet these needs? A systematic integrative review of the literature. BMC Health Services Research, 20(1), 723. 10.1186/s12913-020-05618-3 32767987 PMC7412803

[bibr41-19375867231225395] Sanford A. M. Orrell M. Tolson D. Abbatecola A. M. Arai H. Bauer J. M. Cruz-Jentoft A. J. Dong B. Ga H. Goel A. Hajjar R. Holmerova I. Katz P. R. Koopmans R. T. C. M. Rolland Y. Visvanathan R. Woo J. Morley J. E. Vellas B. (2015). An International Definition for “Nursing Home.” Journal of the American Medical Directors Association, 16(3), 181–184. 10.1016/j.jamda.2014.12.013 25704126

[bibr32-19375867231225395] Schwarz N. Oyserman D. (2001). Asking questions about behavior: Cognition, communication, and questionnaire construction. American Journal of Evaluation, 22(2), 127–160.

[bibr33-19375867231225395] Shreffler J. Huecker M. R. (2023). Types of variables and commonly used statistical designs. StatPearls. http://www.ncbi.nlm.nih.gov/books/NBK557882/ 32491805

[bibr34-19375867231225395] Strudwick G. Hardiker N. R. (2016). Understanding the use of standardized nursing terminology and classification systems in published research: A case study using the international classification for nursing practice(®). International Journal of Medical Informatics, 94, 215–221. 10.1016/j.ijmedinf.2016.06.012 27573329

[bibr35-19375867231225395] Tastan S. Linch G. C. F. Keenan G. M. Stifter J. McKinney D. Fahey L. Lopez K. D. Yao Y. Wilkie D. J. (2014). Evidence for the existing American nurses association-recognized standardized nursing terminologies: A systematic review. International Journal of Nursing Studies, 51(8), 1160–1170. 10.1016/j.ijnurstu.2013.12.004 24412062 PMC4095868

[bibr36-19375867231225395] Törnvall E. Jansson I. (2017). Preliminary evidence for the usefulness of standardized nursing terminologies in different fields of application: A literature review. International Journal of Nursing Knowledge, 28(2), 109–119. 10.1111/2047-3095.12123 26602330

[bibr37-19375867231225395] Ulrich R. S. Berry L. L. Quan X. Parish J. T. (2010). A conceptual framework for the domain of evidence-based design. Health Environments Research & Design Journal, 4(1), 95–114. 10.1177/193758671000400107 21162431

[bibr38-19375867231225395] Waller S. Masterson A. (2015). Designing dementia-friendly hospital environments. Future Hospital Journal, 2(1), 63–68. 10.7861/futurehosp.2-1-63 31098081 PMC6465876

[bibr39-19375867231225395] World Health Organization. (2006). eHealth: Standardized terminology *(* EB118/8; 118th Session). World Health Organization.

[bibr40-19375867231225395] Zhang T. Wu X. Peng G. Zhang Q. Chen L. Cai Z. Ou H. (2021). Effectiveness of standardized nursing terminologies for nursing practice and healthcare outcomes: A systematic review. International Journal of Nursing Knowledge, 32(4), 220–228. 10.1111/2047-3095.12315 33580632

