# Supplementary material for: Are We Speaking the Same Language? Terminology Consistency in EBD
Source: HERD. 2024 Jan 24;17(2):77–96. doi: 10.1177/19375867231225395 (PMC11080391; doi:10.1177/19375867231225395)
Supplement: Supplemental Material, sj-pdf-1-her-10.1177_19375867231225395 - Are We Speaking the Same Language? Terminology Consistency in EBD [file sj-pdf-1-her-10.1177_19375867231225395.pdf]

Table A1: List of Acronyms Used in the Paper

Acronym	Definition
EBD	Evidence-Based Design
AVs	Architectural Variables
HCOs	Health and Care Outcomes
OF	Observed Frequency
EF	Expected Frequency
SD	Standard Deviation

Table A2: Concepts of Architectural Variables (AVs) and their corresponding definitions

Scale-oriented concepts	Definition
Area	The physical size of a space or surface is typically measured in square metres or square feet
Spatial density / capacity	The number of people that can be accommodated within a given space. It is the ratio of available space to the number of occupants
Count of spaces	The number of specific areas or rooms within a building
Attribute-oriented concepts	Definition
Floor covering colour	The colour of the surface material used to cover the floor in a given space
Table setting colour contrast	The degree of contrast between the table's colour and the surrounding space
Floor covering material	The material used to cover the floor in each space
Configuration-oriented concepts	Definition
Outdoor space	The designated area outside a building for recreational, social, or functional purposes
Kitchen	The area in a building used for food preparation and cooking, equipped with appliances and often a dining table
Dining space	The designated area for eating meals, typically located in or adjacent to a kitchen
Common space	The shared area within a building that serves a social or functional purpose, such as a living room or lobby
Bedroom	The private room in a building designed primarily for sleeping, often equipped with a bed and furniture for storage
Family space	The designated area within a building for socializing and spending time with family members or loved ones.
Medical space	The designated area within a building for medical purposes, such as a clinic or examination room.
Personal hygiene space/toilet	The designated area within a building for toileting purposes, often equipped with a toilet and sink
Personal hygiene space/bathroom	The designated area within a building for bathing or showering purposes, often equipped with a shower or tub

Staff space	The designated area within a building for staff or personnel to work, rest, or store equipment or supplies
Circulation system / Spatial configuration	The design and arrangement of the pathways within a building or space, including hallways, corridors, and stairways
Visual connection	The ability to see and make visual contact with other people, spaces, or objects within a building or environment
Physical connection	The ability to physically move and interact with other people, spaces, or objects within a building or environment
Adaptive-oriented concepts	Definition
Indoor temperature	The temperature inside a building or specific area
Noise level	The amount of unwanted sound or noise within a space
Noise source	The origin or cause of unwanted sound or noise within a space
Furniture-oriented concepts	Definition
Furniture type	The specific type or category of furniture used in a space
Furniture character / style	The aesthetic style or design of the furniture in a space
Performance-oriented concepts	Definition
Light level	The level of brightness or illumination within a space
Bright light exposure	The amount or duration of exposure to bright light in a space
Bright light source	The source or origin of bright light in a space
Daylight control	The ability to regulate the amount of natural light entering a space
Visual cue	The visual marker or signal used to guide or direct behaviour or movement within a space
Physical cue	The physical marker or signal used to assist or direct behaviour or movement within a space
Visual barrier	The visual obstruction that impedes visibility within a space
Physical barrier	The physical obstruction or boundary that physically separates different areas or zones within a space
Ambience-oriented concepts	Definition
The possibility of personalizing the environment	The degree to which individuals can modify or adapt a space to their own needs, preferences, or personality
Food service	The ways of providing of meals or food-related services within a space
Gradation of space	The use of changes in scale, height, or other elements to create a sense of depth, hierarchy, or variety within a space
Visual elements	The visual features or aesthetics of a space, including colour, texture, shape, and form
Auditory elements	The auditory features or acoustics of a space, including sound quality, and reverberation
Tactile elements	The tactile or haptic qualities of a space, including the texture, weight, and feel of surfaces or objects
Olfactory elements	The olfactory or scent-related features of a space, including the presence or absence
Overall ambiance atmosphere	The combined effect of all the sensory features of the built environment on the overall mood, comfort, and well-being of people with dementia

Table A3: Concepts of Health and Care Outcomes (HCOs) and their corresponding definitions

Physiological-oriented concepts	Definition
Heart rate	The number of times the heart beats per minute, typically measured as a vital sign to assess cardiovascular health
Blood pressure	The force of blood against the walls of arteries, measured in millimetres of mercury (mmHg) and used to assess cardiovascular health
Body temperature	The degree of heat in the body, typically measured in degrees Fahrenheit (F) or Celsius (C), is used to assess overall health and diagnose illness.
Physiological therapeutic care	Care provided to address physiological or physical health needs, such as medication management, wound care, or rehabilitation therapy
Mortality	The rate or likelihood of death among a specific population or patient group, often used as a measure of healthcare quality
**Physical-oriented concepts**	**Definition**
Physical therapeutic care	Care provided to address physical health needs, such as rehabilitation therapy or pain management
Pain	The unpleasant sensation or emotional experience associated with actual or potential tissue damage, typically measured on a numeric scale and used to assess and manage pain levels
Weight	The measure of a person's body mass, typically measured in pounds (lbs) or kilograms (kg), is used to assess overall health and diagnose illness.
Food intake	The amount and type of food consumed by a person, typically measured in calories or specific nutrients, is used to assess dietary needs and overall health
Fluid intake	The amount and type of fluid consumed by a person, typically measured in ounces or millilitres, is used to assess hydration needs and overall health.
**Psychological-oriented concepts**	**Definition**
Cognitive abilities	The mental processes and functions involved in learning, memory, perception, and decision-making, used to assess overall cognitive health and diagnose cognitive impairments
Cognitive distractions	Environmental factors or stimuli that can interfere with cognitive processes, such as noise, lighting, or other sensory stimuli
Circadian rhythms	The natural 24-hour cycles that regulate sleep-wake cycles, hormonal production, and other physiological processes, used to assess overall health and diagnose sleep disturbances
Sleep disturbance	Disruption or alteration of the natural sleep-wake cycle, often caused by environmental factors, medical conditions, or other factors, and used to assess overall health and diagnose sleep disorders
Psychotropic drug use	The use of medications that affect the mind or behaviour, typically used to treat mental health conditions such as depression, anxiety, or psychosis
Stimulation	Environmental or sensory factors that can promote cognitive, physical, or emotional well-being, used to promote overall health and well-being
Neuropsychiatric symptoms	Symptoms or behaviours associated with mental health conditions, such as depression, anxiety, or psychosis, used to assess and manage mental health needs
**Behaviour-oriented concepts**	**Definition**
Aggressive behaviour	Behaviours or actions that pose a threat or harm to others, used to assess, and manage behavioural health needs

Disruptive behaviour	Behaviours or actions that interfere with daily activities or routines, used to assess and manage behavioural health needs
Agitated behaviour	Restlessness, anxiety, or other behaviours that indicate emotional or psychological distress, used to assess, and manage mental health needs
Confused reaction behaviour	Behaviours or actions that indicate confusion or disorientation, used to assess and manage cognitive health needs
Demeanour-oriented concepts	Definition
Social contact	Interactions with others, including family, friends, and healthcare providers, are used to assess and promote social well-being
Informal social interactions	Unstructured interactions with others, such as casual conversations
Communication skills	The ability of residents to convey their needs and desires effectively and the ability of the staff to understand them
Engagement in activities	The extent to which a resident participates in activities, such as social or recreational activities
Activity of daily life	The ability of a resident to independently perform activities of daily living, such as grooming, bathing, and dressing
Walking performance	The ability of a resident to walk or move around independently, with or without mobility aids
Wayfinding	The ability of a resident to navigate and find their way around the building, including access to services and amenities
Frequency of toilet use	The number of times a resident uses the toilet or requires assistance with toileting within a given period
Motor and process test	The set of tests or assessments used to evaluate a resident's physical abilities such as balance
Human rights-oriented concepts	Definition
Privacy	The ability of residents to control their personal information and physical space, as well as their right to be left alone
Safety	The level of protection and security provided to residents, including protection from accidents and harm
Quality of life	The overall well-being and satisfaction of residents, including their physical, emotional, and social needs
Liberty and autonomy	The ability of residents to make their own decisions and control their own lives, to the extent possible
Personal strengths	The unique strengths, abilities, and qualities of a resident that can be used to promote their well-being and success
